# Strontium isotope evidence for Neanderthal and modern human mobility at the upper and middle palaeolithic site of Fumane Cave (Italy)

**DOI:** 10.1371/journal.pone.0254848

**Published:** 2021-08-24

**Authors:** Michael P. Richards, Marcello A. Mannino, Klervia Jaouen, Alessandro Dozio, Jean-Jacques Hublin, Marco Peresani

**Affiliations:** 1 Department of Archaeology, Simon Fraser University, Burnaby, British Columbia, Canada; 2 Department of Human Evolution, Max Planck Institute for Evolutionary Anthropology, Leipzig, Germany; 3 Department of Archaeology and Heritage Studies, School of Culture and Society, Aarhus University, Højbjerg, Denmark; 4 Géosciences Environnement Toulouse, UMR 5563, CNRS, Observatoire Midi Pyrénées, Toulouse, France; 5 Department of Humanities, Section of Prehistoric and Anthropological Sciences, Ferrara University, Ferrara, Italy; 6 Department of Cultural Heritage, Bologna University, Ravenna, Italy; 7 Institute of Environmental Geology and Geoengineering, National Council of Research, Milano, Italy; University at Buffalo - The State University of New York, UNITED STATES

## Abstract

To investigate the mobility patterns of Neanderthals and modern humans in Europe during the Middle-to-Upper Palaeolithic transition period, we applied strontium isotope analysis to Neanderthal (n = 3) and modern human (n = 2) teeth recovered from the site of Fumane Cave in the Monti Lessini region of Northern Italy. We also measured a large number of environmental samples from the region, to establish a strontium ‘baseline’, and also micromammals (vole teeth) from the levels associated with the hominin teeth. We found that the modern humans and Neanderthals had similar strontium isotope values, and these values match the local baseline values we obtained for the site and the surrounding region. We conclude that both groups were utilizing the local mountainous region where Fumane Cave is situated, and likely the nearby Lessini highlands and Adige plains, and therefore the strontium evidence does not show differening mobility patterns between Neanderthals and modern humans at the Fumane site.

## Introduction

We have little understanding of the mobility patterns of European Neanderthals, and especially if they differed from those of the first modern humans in Europe. Reconstructing mobility in archaeology is especially difficult, as it relies mainly on indirect evidence, such as determining the geological sources of stone tools, the seasons of animal exploitation (i.e. hunting), the provenance of pigments, shells and other materials introduced into the site and the inferred use of the larger landscape by looking at the density of archaeological sites, and the site sizes and uses. These methods have resulted in a plethora of often diametrically opposite interpretations of Neanderthal mobility and territoriality [1–4].

There are more direct measures of human mobility that use hominin skeletal remains, and here we applied one of these methods, strontium isotope ratios in tooth enamel (for reviews see Britton [5] and Bentley [6]). When human teeth are being formed (largely in childhood) occasionally strontium is substituted for calcium in enamel. The strontium is from ingested food and water and has a distinct composition of the various isotopes of strontium, which is measured and reported as the strontium isotope ratio (of the isotopes ^87^Sr compared to ^86^Sr). These strontium isotope ratios of food and water are related ultimately to the strontium isotope ratios of the underlying bedrock where the food was grown, and to the various sources of water. It is possible then to determine, usually by measuring plants and water from a region, a ‘local’ strontium isotope value (‘bioavailable’) that can then be used as a comparison to the enamel strontium value from the human [7]. Using this method, it is then possible to determine if the tooth formed when the human was consuming food and water locally or lived elsewhere during childhood tooth formation. This method has now been extensively used in archaeology to determine if individuals were ‘local’ or ‘non-local’ and with more detailed understanding of the baseline strontium variation in a region it is also sometimes possible to suggest a place of origin.

### Grotta di Fumane (Fumane Cave)

**Fig 1:**
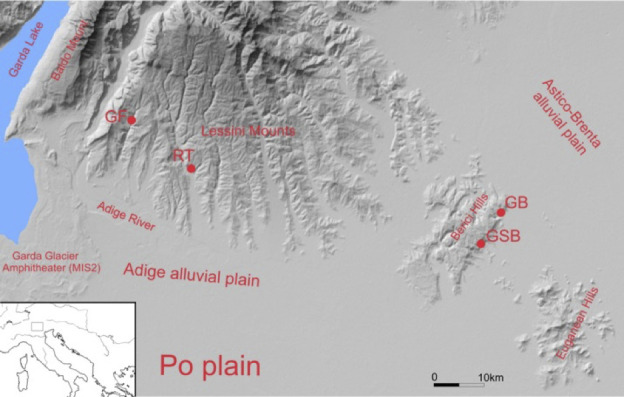
Location of Fumane Cave (GF) and other middle and upper paleolithic sites in NE Italy. (RT = Riparo Tagliente; GB = Grotta Broion; GSB = Grotta di San Bernardino). The map was produced with the CorelDRAW12 software, using digital terrestrial models (DTM) downloaded from http://www.regione.veneto.it/web/ambiente-e-territorio/geoportale and from http://www.territorio.provincia.tn.it/portal/server.pt/community/portale_geocartografico_trentino. The figure is similar but not identical to the original image and is therefore for illustrative purposes only.

We had the opportunity to be able to use strontium isotope analysis to compare the mobility of late Neanderthals and early European modern humans (hereafter referred to as Anatomically Modern Humans, AMH) at the site of Grotta di Fumane (Fumane Cave), where teeth of Late Pleistocene hominins have been found in close stratigraphic succession [8, 9]. The cave is located in the western part of the Monti Lessini (Lessini Mountains), a fan-shaped plateau in the Venetian Pre-Alps reaching up to 1850 m a.s.l. and intensively settled during the Lower and Middle Palaeolithic [10] (**Fig 1**).

Fumane Cave is a key site for understanding the Middle-to-Upper Paleolithic transition in Italy, with a sequence spanning from the Mousterian, through the Uluzzian and Proto-Aurignacian [11–13]. Excavations at the site started in 1998 and are still ongoing under the aegis of the Italian Ministry of Cultural Heritage and the direction of the University of Ferrara. Mousterian industries are found in units A12-A11, A10, A9, A6-A5, A4, followed by the Uluzzian in A3, and the Proto-Aurignacian in A2-A1 and D3. With the exception of units A12-A11 and A10, the cultural levels were investigated over areas varying in size from 6 to 75 square meters. Lithics and faunal remains were densely scattered over the living floors, particularly in the case of levels A11, A10, A9, A6 and A2-A1 [11, 14]. The lithic techno-complexes were produced with raw material mostly provisioned within a 15km radius from the site both by Neanderthals and modern humans [15]. Considerable changes in material culture have been documented through the sequence at Fumane. These include the development of a symbolic repertoire by the late Neanderthals who occupied the cave [16–18] and that was shared by conspecifics across the whole distribution of these humans. The most significant changes in material culture, however, occurred with the start of the Uluzzian around 45.0 ka cal BP [13, 19] followed by the modern human occupation with the Proto-Aurignacian (between 41.1 ka cal BP and 38.5 ka cal BP) [20], when painted stones [21] and large assemblages of stone and bone tools and ornaments on marine shells collected up to 300km far from the cave [15, 22, 23] made their first appearance.

Fumane Cave is located 350 m above modern sea level (a.m.s.l.). To the west of Fumane, the Monti Lessini plateau ends at the Adige Valley, a long and deep cut connecting the inner Alpine region with the Po Plain (**Fig 1**). The immediate surroundings of the cave feature several morpho-tectonic terraces connected to the bottom of the Fumane valley by steep slopes and rock walls with many caves and shelters. The cave is, thus, placed in a strategic position that allowed its occupants access both to the Lessini highlands and the Adige and Po plain below. The eastern Italian Alps are a physical and environmental threshold, which in the Middle Paleolithic would have been characterized by ice fields and alpine glaciers during cold stages and temperate vegetation during warm phases [24].

The sedimentary rocks that dominate the Monti Lessini are composed mainly by Mesozoic limestones and dolostones. The main succession includes, in stratigraphic order, from the most ancient to the most recent: the Dolomia Principale Triassic dolostone bedrock, the Calcari Grigi limestones (Jurassic), the oolitic limestones of San Vigilio (Jurassic), the Rosso Ammonitico nodular limestones (Jurassic), the Scaglia Variegata marly limestones (Cretaceous), the Scaglia Rossa marly limestones (Cretaceous), Eocene limestones, basalt and tuff, Miocene calcarenites and Quaternary glacial and fluvial sediments [25]. It is therefore most likely that the local strontium isotope composition will mainly reflect the composition of the Jurassic and Cretaceous limestone formations.

Strontium isotope analyses on deciduous human teeth of the Mousterian (Fumane 1, 4 and 5) and Proto-Aurignacian (Fumane 2) cultures, and on a permanent molar from the transitional layer (Fumane 6), allowed us to investigate mobility between 47.6–45.0 ka cal BP and 41.1–38.5 ka cal BP [9], the period when Neanderthals were replaced by modern humans in this region (Table 1).

**Table 1:** Summary data of the five hominin teeth measured in this study.

Individual	Period	Level	Element	Approximate age of crown formation (in months)	Wear stage
Fumane 2	Proto-Aurignacian	A2	Rdi^2^	-2.0 to 0/4.5	6
Fumane 6	Uluzzian/PA	A3I	pM	1.5 to 30	5
Fumane 4	Mousterian	A9I	Rdi^1^	-2.0 to 0/1.5	6
Fumane 5	Mousterian	A9	Rdi_2_	-2.0 to 0/4.5	4
Fumane 1	Mousterian	A11	Ldm_2_	-1.0 to 10.5/18	5

Attributions to Anatomically Modern Humans (AMH) or Neanderthals (N) are based on Benazzi et al.^8^. Further descriptions of the teeth are given in the SI. The wear stages are based on a study of tooth wear on *Homo sapiens* specimens from North America [33], given that similar estimates are not available for Neanderthals.

## Materials

### Bioavailable strontium baseline

**Fig 2:**
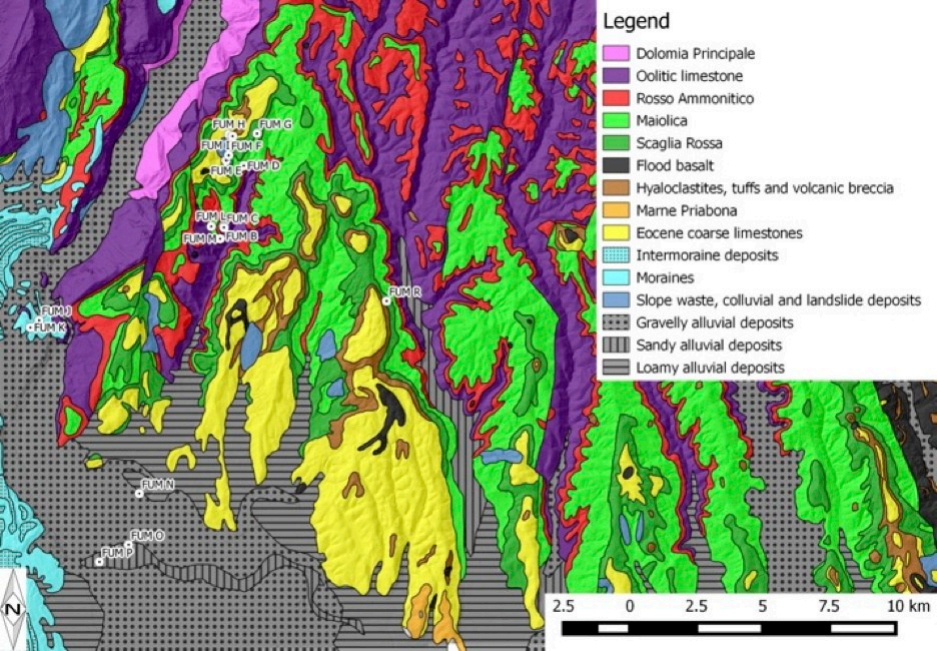
Geology of the monti lessini and nearby areas of veneto with the localities sampled for the strontium isotope baseline. Geo-topographic data have been downloaded from http://www.regione.veneto.it/web/ambiente-e-territorio/geoportale and http://www.territorio.provincia.tn.it/portal/server.pt/community/portale_geocartografico_trentino. These have been elaborated using open source QGIS 2.4 Chugiak (Quantum GIS) software available at http://www.qgis.org/it/site/ and adopting Monte Mario / Italy zone 1 as reference system. The figure is similar but not identical to the original image and is therefore for illustrative purposes only.

The study of human mobility through strontium isotope analyses requires knowledge of the isotopic composition of the geological formations close to a site, to establish if an individual had strontium isotope values consistent with the local environment or not [26, 27]. Understanding the local geology where a strontium isotope study is undertaken is important, however the strontium values in biological organisms are not directly related to the strontium values of the geological strata. Instead they reflect ‘bioavailable’ strontium and a method to determine the bioavalble strontium values for a region are to measure the strontium in modern organisms like plants and molluscs, taken from the different lithologies around the site [7]. These measurements then represent the local biologically-available isotope signature and constitute the ‘strontium isotope baseline’. To achieve this, we collected modern plants and shells of terrestrial molluscs at 19 localities mapped in **Fig 2**, as detailed in the Methods section below and listed in **S1 Table 1 in S1 File**. The sampling of the strontium isotope composition of the local geology targeted all the main lithologies found in and around the Monti Lessini, extending to the Euganean Hills, where Neanderthals moved from Monti Lessini [28]. This coverage is sufficient to ascertain whether the isotope data on the human teeth are indicative of a local origin from around the cave and/or of movements uphill from it, across the Po Plain, or further afield.

### Archaeological tooth enamel samples

In addition to the environmental bioavailable baseline samples, we also measured the strontium isotope values of sixteen molars of *Microtus arvalis* (common vole) taken from the same stratigraphic units as the human teeth [29]. These strontium isotope analyses on rodent enamel were undertaken to provide additional information for the strontium baseline as they are contemporary with the homins, so would have recorded at the bioavailable strontium value at the different times the site was occupied (and teeth were deposited in the strata). *M*. *arvalis* lives in a wide range of open habitats including meadows, forest steppe, moist forested areas and agricultural land. This species was selected as a proxy for the local strontium isotope baseline, because it has restricted home range sizes [30], which vary between 125 m^2^ in open vegetation (e.g. in wildflower strips) and 1500m^2^ in forested areas (e.g. in pine plantations). We selected teeth that did not have evidence of digestion by raptors [29, 31].

In total five hominin teeth were found at Fumane Cave [8], as shown in **Table 1** and described in further detail in the Supplementary Information. Three of the teeth, labelled Fumane 1, 4 and 5 (sample codes FUM1, FUM4 and FUM5), are deciduous teeth attributed to Neanderthals originating respectively from the late Mousterian units A11 (Levallois technology) [12], A9I and A9 (Discoid technology) [7]. The chronology of A9 is based on a single reliable date of 47.6–45.0 ka cal BP [20], which corresponds to the time when the last Neanderthals were still present in Italy [32].

A small human permanent tooth fragment was recovered in unit A3I and labelled Fumane 6 (sample code: FUM6). This part of the stratigraphic sequence dates from 43.4 to 40.3 ka cal BP and is attributed to the early Upper Paleolithic culture known as the Uluzzian^13^. However, given these layers were subject to post-depositional disturbance in the easternmost area of the cave entrance and that a dispersion of Aurignacian lithics from unit A2 into unit A3 occurred, Fumane 6 may be Proto-Aurignacian [8, 13]. In this study we refer to this tooth as tentatively being from an AMH.

The fifth tooth (Fumane 2: sample code FUM2) was recovered in unit A2 and attributed to AMH [9]. At the largest confidence interval, unit A2 has been dated to between 41.1 ka cal BP and 38.5 ka cal BP, which means that Fumane 2 is the oldest human remain associated with an Aurignacian-related Upper Paleolithic culture in Europe.

The teeth from Fumane belong to children, with the possible exception of Fumane 6 (which, given the uncertainty in classification, may have formed from the first/second or third year of life up to 17.5 years [34]). In the case of the four individuals that were in their childhood (Fumane 1, 2, 4 and 5), tooth formation (amelogenesis) would have occurred between late intrauterine life and from the first month and a half up to 18 months after birth, so largely prior to walking age and weaning [35]. It should be added that all the human teeth from Fumane, analyzed in this study, display advanced stages of wear (**Table 1**) [8, 9], which suggests that their enamel was fully mineralized and, thus, their strontium isotope composition potentially reflects the entire mineralization time of the crown.

## Methods

### Strontium isotope baseline for modern plants and snails

The first three localities sampled to establish the strontium isotope baseline are around Fumane Cave and represent the strontium isotope composition for the immediate vicinity of the cave (**Fig 2 and S1 Table 1 in S1 File**). Most of the other samples were collected from the geological formations present in the Monti Lessini, following the geological stratigraphic succession described above (**S1 Table 1 in S1 File**). Our sampling did not include the easternmost part of the Monti Lessini characterized by the basalts of the Castelvero/Schio basin, although basaltic lithologies were sampled in the Euganean Hills (i.e. FUM-PA and FUM-Q). Additional samples were taken in the morainic deposits of the Rivoli ‘amphitheatre’ at the entrance of the Adige valley and in the alluvial plain of the Adige River. The sampling localities were chosen by avoiding areas close to agricultural land where fertilizers may have been sprayed, which affect strontium isotope compositions of organisms [7], and by selecting outcropping bedrock formations near forests and quarries. The exact location of the sampling spot was recorded using a hand-held GPS device (**S1 Table 1 in S1 File**). Sampling at each locality aimed to acquire leaves of plants with shallow roots (SRP: grasses), medium-rooting plants (MRP: small shrubs) and deep-rooting plants (DRP: large shrubs or small trees). Snail shells almost only of the terrestrial gastropod *Pomatias elegans* were also sampled.

### Sample pretreatment and analyses

Modern plants and molluscs, and archaeological common vole teeth were prepared and analyzed for solution strontium isotope measurements, whereas the strontium isotope compositions of the human teeth were assessed using laser ablation (which was necessary, rather than invasive drilling for solution chemistry pretreatment, due to the rare and fragile nature of the specimens and to preserve the integrity of the specimens). All the analyses were conducted in the laboratories of the Department of Human Evolution at the Max Planck Institute for Evolutionary Anthropology (MPI-EVA) in Leipzig, Germany. All necessary permits were obtained for the described study, which complied with all relevant regulations.

### Solution strontium isotope analyses

The preparation protocol for plants, snails and vole teeth samples is described by Hartman and Richards [36] and includes a column chromatography step outlined by Deniel and Pin [37]. A Thermo Fisher Neptune MC-ICP-MS instrument (see **S1 Table 2 in S1 File** for operational parameters) was used to measure the Sr isotope ratios. The reference standard SRM 1486 and one blank were quantified in parallel for each run of thirteen samples. A mean ^87^Sr/^86^Sr ratio of 0.70932±0.00003 (n  =  5) resulted from the systematic measurements of the reference standard, which is consistent with the long-term measurements performed in this laboratory 0.70930±0.00003 (n  =  94). The international standard SRM 987 yielded a mean ^87^Sr/^86^Sr ratio of 0.710278±0.000005 (n  =  31), and the offset with the accepted value of 0.71024±0.00004 was used to correct the isotope ratios [38, 39]. Strontium concentrations were estimated using the ^88^Sr signal intensities (V) of three SRM 987 standards with different concentrations (100, 400 and 700 ppb), following the protocol outlined in Copeland et al. [40].

### Laser ablation strontium isotope analyses

All teeth were analysed using a 193 nm laser ablation unit (Analyte Excite, Photon Machine) coupled to the same MC-ICP-MS used for solution analyses described above. Operational parameters are described in **S1 Table 2 in S1 File**. The isobaric interference of ^87^Rb was corrected using the ^85^Rb signal. Corrections were also made to account for the presence of Kr in the carrier gas using ^83^Kr. Blanks were quantified prior to each measurement when the laser was switched off. ^89^Y was monitored for all samples to assess potential REE contributions. Careful attention was paid to perform the measurements on a flat surface of enamel to avoid potential isotopic fractionation due to unfocused laser beam, even though these effects have been shown not to be significant [41]. Prior to each analysis, a pre-ablation along the path of the analysis was performed to remove potential surface contamination. The number of laser ablation tracks measured per tooth varied, depending on tooth integrity, between 8 for intact specimens (Fumane 1 and 2) and 2 for fragmentary ones (Fumane 4, 5 and 6) (**S1 Table 3 in S1 File**). For each of the teeth two measurements were made on the outer enamel (see SI for a schematic representation of where the sample tracks were situated on each tooth). For two teeth (Fumane 1 and Fumane 2) we were also able to measure a cross section of the enamel (inner enamel) as these teeth were sectioned for other analyses. Considering that it is not possible to establish the time period covered by each track of laser-ablated enamel, especially for the outer enamel, as shown by Montgomery et al. [42], we did not to speculate on possible areas over which the specific individuals may have moved based on differences within the laser ablation tracks and instead report the average value of all of the individual laser ablation spots within a single raster measurement. The results for each track are given in **S1 Table 3 in S1 File**, and the average of the values for the measured tracks for each tooth are given in **Table 2**.

**Table 2:** Strontium isotope data for hominin teeth from Fumane Cave.

Individual	Attribution	87Sr/86Sr	1 SD	Number of transects
Fumane 1	N	0.7087	0.0011	6
Fumane 2	AMH	0.7090	0.0011	8
Fumane 4	N	0.7107	0.0013	2
Fumane 5	N	0.7103	0.0004	2
Fumane 6	AMH	0.7082	0.0006	2

Measurements were made on the outer portions of enamel (external) for the five teeth (see SI for schematic representation of where the strontium transects were measured) and also on internal cross-sections for Fumane 1 and 2. Measurements reported here are the average of two transects for each tooth. Additional information on the measurements is provided in **S1 Table 3 in S1 File**.

Two internal standards, an archaeological cattle tooth (FBC4M) and a reindeer tooth (*Rangifer tarandus*) (NCH3) were measured alongside (bracketing) the archaeological specimens in each sample run. These two internal standards have ^87^Sr/^86^Sr values of 0.7115 (FBC4M) and 0.7063 (NCH3) from solutions measured on both TIMS and MC-ICP-MS [43]. The average LA-ICP-MS (uncorrected) results for these two produced results that were similar to the solution values, FBC4M = 0.7127±0.0002 (1σ, *n* = 8) and NCH3 = 0.7066±0.0003 (1σ, *n* = 8).

It has been observed by some researchers that especially for enamel bioapatite with relatively low strontium concentrations there may be some interference at mass 87 from calcium (^40^Ca^31^P^16^O) or argon (^40^Ar^31^P^16^O) polyatomic compounds produced during the ablation process [44]. Therefore, most researchers check for this potential interference and if detected, correct the values to take this into account. This is done either by bracketing their samples with a matrix-matched standard and correcting based on the measured difference in the standard from the laser ablation compared to the solution strontium value [45, 46]. Or by producing a correction curve using matrix matched standards where the difference between the solution values for those standards and the measured laser ablation values is plotted against the strontium concentration [47, 48].

We have observed in this study, a previous study of a Neanderthal tooth (Richards et al. [49]), and in unpublished laser ablation measurements that Neanderthal teeth often have low strontium concentrations compared to modern humans and fauna from the same site. This could be because of the high trophic level of Neanderthal diets [50, 51], as the relative amounts of strontium in bones and teeth of carnivores are much lower than herbivores within the same ecosystem [52, 53]. In our study we found that the two modern humans (FUM2, FUM6) had higher enamel strontium concentrations than the three Neanderthal teeth, and in the case of the inner enamel of FUM1, it was 3 times higher than the average strontium concentration of the three Neanderthals (**S1 Table 3 in S1 File**).

Therefore, as we had relatively low strontium concentrations for the Neanderthals in our study, we chose to apply a correction to account for potential interference from the ablation processes. We used the method outlined in Horstwood et al. [47] and more recently applied by Lugli [54] and Lugli et al. [48]. We produced a calibration using the slope of the regression line of the standards calculated from plotting the relative difference between the solution and laser ablation 86Sr/87Sr values against measured 1/88Sr (as a measure of strontium concentration). The corrected values are reported in Tables 2 and 3 and the uncorrected values are given in **S1 Table 3 in S1 File**. The average difference in values for the modern human and Neanderthal laser ablation measurements between the uncorrected and corrected ^87^Sr/^86^Sr ratios was 0.0026. The ^84^Sr/^86^Sr ratio, which is established as 0.0565 was also measured for these samples, although it has limited utility as a quality indicator [43, 44], and the values for each ablation track are given in the **S1 Table 3 in S1 File**. The average measured ^84^Sr/^86^Sr value for all of the archaeological tooth samples is 0.0563±0.0008 (1σ, *n* = 20).

## Results

### Modern plants and snails for the strontium isotope baseline

**Fig 3:**
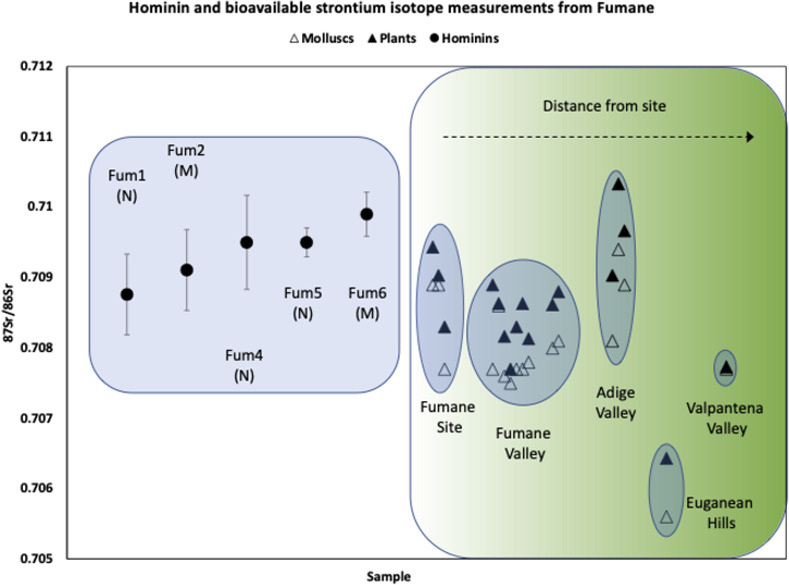
Strontium isotope values of hominin dental enamel and local molluscs and plants from the fumane site and surrounding regions. The plant and mollusc data is given in the Supplementary Information. The Fumane 1 and Fumane 2 values are the average of the outer and inner enamel measurements for each of the two teeth. The Fumane 4, 5 and 6 values are the average of two outer enamel measurements. Error bars on the hominins are given as 1s, and the 1s errors on plant and snail strontium measurements are much smaller, and plot within the symbols.

The strontium isotope ratios of the plants for the modern baseline from the immediate vicinity (i.e. within a few hundred metres) of Fumane Cave (0.7092±0.0006; 2σ), as well as 1km (0.7087±0.0005; 2σ) and 5km (0.7084±0.0006; 2σ) upland from it, are typical of Jurassic and Cretaceous limestones and strongly overlap (**Fig 3 and Table 3** and **S1 Table 4 in S1 File**). A slight trend towards less radiogenic values is present moving from the cave up to the Lessini plateau, whilst the opposite is the case moving towards the Adige Valley and the Po Plain. The strontium isotope ratios of the snails generally overlap with those of the plants (**Table 3** and **S1 Table 4 and 5 in S1 File**).

**Table 3:** Strontium baseline isotope data for the Fumane site and nearby regions based on modern plants, modern land snails and archaeological teeth of *Microtus arvalis* (common vole).

Samples	Site	1km radius	5km radius
Plants	0.7090±0.0005	0.7087±0.0005	0.7084±0.0006
Snails	0.7085±0.0007	0.7082±0.0006	0.7079±0.0005
*M*. *arvalis* (mean)	0.7096 ± 0.0012		

The localities representative of the lithology at the site of Fumane Cave are FUM-A, FUM-B and FUM-C. These localities and FUM-L and FUM-M are part of the 1km radius cluster, while localities FUM-D, FUM-E, FUM-F, FUM-G, FUM-H and FUM-I are included in the 5km radius cluster. Locations of these sampling locales are given in **S1 Table 1 in S1 File**.

### Common vole teeth

The strontium isotope values of the 16 molars of *Microtus arvalis* sampled from the four stratigraphic units of origin of the human teeth are relatively homogeneous (**S1 Table 6 in S1 File**), with the following means and standard deviations (2σ): A11 = 0.7096±0.0008; A9 = 0.7095±0.0021; A3 = 0.7099±0.0004 and A2 = 0.7095±0.0007. The overall mean for the common vole teeth (0.7096±0.0012) overlaps with those of the plants collected at the site and within a 1km radius from it, as well as with the land snails collected near the cave, indicating that the isotopic composition of this rodent is a good proxy for the isotopic composition of the lithologies around Fumane at the time of occupation and deposition of the hominin teeth. Moreover, this suggests that diagenesis is unlikely to have occurred or, at least, not to have affected dental enamel enough to shift isotopic compositions significantly away from that of the local baseline range. Diagenesis can probably also be excluded because, as in other studies of archaeological rodent enamel^36^, there is no significant correlation between ^87^Sr/^86^Sr ratios and strontium concentration (R^2^ = 0.0064) nor between ^87^Sr/^86^Sr ratios and strontium concentration expressed as 1/Sr ppm (R^2^ = 0.0685).

### Hominin teeth

The strontium isotope composition of the hominin dental enamel from Fumane overlaps with values we measured from modern plants and snails from near to the site and from the surrounding region (**Fig 3** and **Tables 2 and 3**). They are also very similar to the values from the archaeological common vole (*Microtus arvalis*) teeth associated with the individual teeth (**S1 Table 6 in S1 File**).

### Neanderthal teeth (Fumane 1, 4 and 5)

We obtained seven measurements of the strontium isotope values of the outer and inner enamel of Fumane 1 (0.7087±0011). This value is lower (but within 1σ) of the measured vole samples from the same layer (Layer A11, 0.7096±0.0004). The Fumane 4 Neanderthal has an outer enamel strontium value of 0.7107±0.0013, which is higher (but within 1σ) than the contemporary archaeological vole teeth from the same archaeological context (Layer A9, 0.7095±0.0010). This same pattern is also observed for the third Neanderthal tooth, Fumane 5, which had outer enamel strontium values of (0.7103±0.0004) which was also higher than the contemporary vole teeth (Layer A9, 0.7095±0.0010). As the Fumane 4 and 5 specimens have similar mean strontium isotope values, this may indicate that they belong to the same individual, which was hypothesized previously^8^ due to their identical stratigraphic unit of origin and developmental stage.

### Anatomically modern human teeth

The AMH (Aurignacian) Fumane 2 tooth had an average enamel (based on 2 outer and 6 inner measurements) strontium value of 0.7090±0.0011). These values are very similar to the vole teeth from the same context (Layer A2, 0.7095±0.0003). Fumane 6, which is here tentatively classified as belonging to an AMH has an outer enamel strontium value of 0.7082±0.0006, which has a lower value (not overlapping at 2σ) than the associated vole teeth from the same archaeological context (Layer A3, 0.7099±0.0002).

## Discussion

As tooth enamel strontium is incorporated during tooth formation, and the Neanderthal and modern human teeth (with the exception of Fumane 6, the Uluzzian individual) are from children, the strontium values will reflect both the diet of the child as well as that of the mother, especially for enamel that formed *in utero* or during breastfeeding. With the exception of Fumane 6, all crowns analysed in this study, started growing *in utero* and the formation of three of these (i.e. the deciduous incisors) occurred for at least around a third before birth. These two Neanderthal (Fumane 4 and Fumane 5) and one modern human specimen (Fumane 2) may, thus, have incorporated relatively large proportions of strontium from the mother’s plasma. If the prevailing proportion of strontium originated from skeletal stores, it thus would mean that the strontium isotope composition of Fumane 4 and Fumane 5 is informing us on the lithologies of origin of the mother. Therefore, in our dicussions below it is important to note that the mobility of these individuals likely reflects their own mobility and that of their mothers.

We have plotted the hominin strontium values compared to the mollusc and plant values from the different regions at and around the Fumane site (**Fig 3**). As can be seen, the plant and mollusc values from the site, the Fumane valley and the Rivoli amphitheatre are similar, and generally cluster between 0.7075 and 0.7095. The values from the Adige plain are similar, however some samples are more radiogenic, while the samples furthest from the site, from the Euganean hills and the Valpantena valley are less radiogenic.

As can be seen in **Fig 3**, the hominins all have values that fall within the range of bioavailable samples from the Fumane Cave site and surrounding valley, and most of them also overlap with values from the Adige plain. However, they do not overlap with the bioavailable samples from either the Euganean hills or the Valpantena valley. These data show that the hominins, both modern humans and Neanderthals, have values that can be interpreted as showing that both were living in, and moving around, the region local to the Fumane site when their enamel was formed, including likely away from the cave and valley onto the Adige plain.

### Other indicators of mobility

The strontium isotope data presented here shows that the Neanderthal and AMH teeth formed when these individuals (or their mothers) were in areas around the Fumane site. As noted above, this represents a relatively short (less than a year) in the life of these individuals, so if larger scale movements occurred at other times of life, we would not be able to see them in the strontium values. Indeed, there are other indicators of longer-distance mobility by Neanderthals at the site. Of note is the presence of fossil marine shell of *Aspa marginata* recovered in stratigraphic unit A9 [17]. Apart from two scrapers made of flint from the Rosso ad Aptici formation outcropping West of the Garda Lake [55], the ochered *Aspa marginata* is the only ‘exotic object’ from the Mousterian levels and it was found in the same level as Fumane 4 and 5. This shell was collected either from an outcrop south of the Po Plain at the foot of the Apennines, more than 100km away as the crow flies, or along the northern edges of the Po Plain, between 50km and 180km away.

Excluding a handful of bladelets made of radiolarite provisioned west of Lake Garda, the lithic raw material used by the Aurignacians was generally procured within 15km from the site [15]. Most significant habitation features at the site are Aurignacian^11^, but this was also the time when most ‘exotic objects’ were introduced. These are shell ornaments made of Mediterranean marine mollusc taxa, which attest contacts with groups that lived close to the Adriatic or Ligurian seashores by the Fumane modern humans of at least 3-400km and back [23]. Seasonal data obtained studying the formation and growth of ungulate teeth show that the first Aurignacians hunted around Fumane mainly from the end of spring to the end of autumn [15, 56].

A recent study of the trace element composition of three Neanderthal teeth and one Upper Palaeolithic tooth from sites in north-eastern Italy has shown detailed information on weaning patterns for these individual teeth [57]. This study focused on using laser ablation on sections of the teeth at the enamel-dentine junction. The laser ablation measured continuous values of these sections, and also included strontium isotope measurements of small sections of the inner part of two of the Fumane teeth that we also measured here. The values reported in Nava et al. [57] for Fumane 1 (0.7093) and Fumane 2 (0.7088) are similar (within 1σ) of our strontium laser ablation measurements for the same teeth (**Table 2**). Nava et al. [57], based on comparisons with strontium measurements of archaeological vole teeth from Fumane, argue that the modern human (Fumane 2) was in a location distant from the site when their enamel was being formed. This conclusion was made without knowledge of the bioavailable strontium values for the site and surrounding regions. With the environmental strontium data presented here we can clearly see that the values for the two teeth obtained by Nava et al. [57] fall within the range of local strontium values.

## Conclusions

We have presented here strontium isotope data on Neanderthal and modern human teeth, as well as associated fauna and modern plants, from the region around the Middle and Upper Palaeolithic site of Fumane Cave, in Northern Italy. Our studies showed that both the Neanderthal teeth and the modern humans have strontium isotope values that fall within the observed range of modern plant and mollusc values from the site and surrounding region. They also are within the range of values observed for archaeological vole teeth taken from the same levels at the site as the hominin teeth. We therefore interpret this as indicating that both Neanderthals and modern humans utilized resources at and around the cave during the periods of enamel formation (childhood, and potentially *in utero*). This study adds to our growing understanding of mobility in Middle and Late Palaeolithic Europe, and also provides new baseline regional bioavailable strontium data for future researchers.

## Supporting information

S1 File Text and tables. (DOCX)

S1 Fig Schematic drawings of the human teeth sampled for this study with the areas that were laser ablated. (TIF)
