# Correction: Strontium isotope evidence for Neanderthal and modern human mobility at the upper and middle palaeolithic site of Fumane Cave (Italy)

**DOI:** 10.1371/journal.pone.0314425

**Published:** 2024-11-20

**Authors:** Michael P. Richards, Marcello A. Mannino, Klervia Jaouen, Alessandro Dozio, Jean-Jacques Hublin, Marco Peresani

The affiliation for the sixth author is incorrect. The correct affiliation is not indicated. Marco Peresani is not affiliated with #6 but with: Department of Humanities, Section of Prehistoric and Anthropological Sciences, Ferrara University, 44100 Ferrara, Italy.
