# Supplementary material for: Strontium isotope evidence for Neanderthal and modern human mobility at the upper and middle palaeolithic site of Fumane Cave (Italy)
Source: PLoS One. 2021 Aug 24;16(8):e0254848. doi: 10.1371/journal.pone.0254848 (PMC8384160; doi:10.1371/journal.pone.0254848)
Supplement: S1 File — (DOCX) [file pone.0254848.s001.docx]

**S1 Supplementary INFORMATION**

for

**Strontium isotope evidence for Neanderthal and Modern Human mobility at Fumane Cave (Italy) at the Middle-to-Upper Palaeolithic transition**

Richards, M.P.^1,2^, Marcello A. Mannino^3,2^, Klervia Jaouen^4,2^, Alessandro Dozio^5^, Jean-Jacques Hublin^2^, Marco Peresani^6,7^

^1^ Department of Archaeology, Simon Fraser University, Burnaby, BC V5A 1S6, British Columbia, Canada

^2^ Department of Human Evolution, Max Planck Institute for Evolutionary Anthropology, 04103 Leipzig, Germany

^3^ Department of Archaeology and Heritage Studies, School of Culture and Society, Aarhus University, 8270 Højbjerg, Denmark

^4^ Géosciences Environnement Toulouse, UMR 5563, CNRS, Observatoire Midi Pyrénées, 31400 Toulouse, France

^5^ Department of Humanities, Section of Prehistoric and Anthropological Sciences, Ferrara University, 44100 Ferrara, Italy

^6^ Department of Cultural Heritage, Bologna University, 48121 Ravenna, Italy

^7^ Institute of Environmental Geology and Geoengineering, National Council of Research, Piazza della Scienza 1, 20126 Milano, Italy

**S1 Table 1. Localities across the modern landscape where modern plants and snails were sampled.** These localities are mapped in Fig. 2.

| **Location** | **Position** | **Geographic coordinates** | **Bedrock** |
| --- | --- | --- | --- |
| FUM-A | Fumane valley | N 45° 35’ 52.7’’ E 10° 54’ 29.2’’ | Calcari Oolitici |
| FUM-B | Fumane valley | N 45° 35’ 51.4’’ E 10° 54’ 30.6’’ | Calcari Oolitici |
| FUM-C | Fumane valley | N 45° 35’ 30.3’’ E 10° 54’ 19.3’’ | Calcari Oolitici |
| FUM-D | Fumane valley | N 45° 36’ 45.1’’ E 10° 54’ 56.3’’ | Maiolica |
| FUM-E | Fumane valley | N 45° 36’ 51.9’’ E 10° 54’ 25.8’’ | Scaglia Rossa? |
| FUM-F | Fumane valley | N 45° 36’ 58.1’’ E 10° 54’ 30.4’’ | Scaglia Rossa |
| FUM-G | Fumane valley | N 45° 37’ 23.9’’ E 10° 55’ 20.6’’ | Scaglia Rossa |
| FUM-H | Fumane valley | N 45° 37’ 23.5’’ E 10° 54’ 32.0’’ | vulcanites? |
| FUM-I | Fumane valley | N 45° 37’ 21.3’’ E 10° 54’ 38.1’’ | Calcari Eocenici |
| FUM-J | Rivoli amphitheatre | N 45° 33’ 42.3’’ E 10° 48’ 54.9’’ | glacial deposit |
| FUM-K | Rivoli amphitheatre | N 45° 33’ 33.8’’ E 10° 48’ 39.5’’ | glacial deposit |
| FUM-L | Fumane valley | N 45° 35’ 31.9’’ E 10° 53’ 57.3’’ | Rosso Ammonitico |
| FUM-M | Fumane valley | N 45° 35’ 16.6’’ E 10° 54’ 12.1’’ | Rosso Ammonitico |
| FUM-N | Adige plain | N 45° 30’ 8.4’’ E 10° 51’ 41.6’’ | gravelly alluvial deposit |
| FUM-O | Adige plain | N 45° 29’ 6.3’’ E 10° 51’ 21.0’’ | sandy alluvial deposit |
| FUM-P | Adige plain | N 45° 28’ 46.2’’ E 10° 50’ 29.9’’ | gravelly alluvial deposit |
| FUM-PA | Euganean hills | N 45° 18’ 48.9’’ E 11° 42’ 8.8’’ | Colli Euganei basalts |
| FUM-Q | Euganean hills | N 45° 18’ 11.9’’ E 11° 41’ 14.6’’ | Colli Euganei basalts |
| FUM-R | Valpantena valley | N 45° 33’ 41.6’’ E 10° 59’ 30.3’’ | Scaglia Rossa |

**S1 Table 2. Settings for the MC-ICP-MS Thermo Neptune and the LA Excite system for both solution and laser isotope analyses.**

| **Solution** | |
| --- | --- |
| MC-ICP-MS | Thermo Fisher Neptune™ |
| RF power | 1300 W |
| Cool gas flow rate | 15 L/min |
| Aux gas flow rate | 0.8 L/min |
| Sample gas | 1.21 L/min |
| Interface cones | Ni |
| Mass resolution | Low |
| Lens settings | Optimized for maximum signal intensity |
| Nebulizer | Elemental Scientific Inc., Microflow 100 µL/min, perfluoroalkoxy (PFA) |
| Sensitivity on ^88^Sr | 50 V/ppm |
| Cup configuration | L4 (^82^Kr); L3 (^83^Kr); L2 (^84^Sr); L1 (^85^Rb); Ax (^86^Sr); H1 (^87^Sr); H2 (^88^Sr |
| Data collection | block, 50 cycles, 2 s integrations |
|  | |
| **Laser ablation** | |
| Laser | Excite 193 nm LA system Photon Machine |
| Cell type | Helex chamber |
| LA pulse width | <4 ns |
| He carrier MFC1 flow [L min^−1^] | 0.54 |
| He carrier MFC2 flow [L min^−1^] | 0.560 |
| Maximal fluence [J cm^−1^] | 15.2 |
| Delivered fl. [% of max. fl.] | 70–100 |
| Raster scan lengths [μm] | 500–600 |
| Scan speed [μm s^−1^] | 2 |
| Repetition rate [Hz] | 25 |
| Washout time [s] | 30 |
| *Pre-ablation* | |
| Frequency | 25 |
| Translation rate | 100 µm/s |
| Beam width | 150 µm |
| *Ablation* | |
| Frequency | 25 |
| Translation rate | 2 µm/s |
| Beam width | 150 µm |

**S1 Table 3. ^87^Sr/^86^Sr ratios for the different transects performed on dental enamel by laser ablating the human teeth from Fumane.** Values are the averages of the individual raster measurements made along a single laser ablation transect. N=Neanderthal and M=modern human. Outer = laser ablation transects measured on the outer enamel. Inner = laser ablation transects measured on cross sections of the inner enamel.

| **Specimen** | **Transect** | ***87Sr/86Sr Uncorrected*** | **87Sr/86Sr Corrected** | **1 SE** | **89Y (V)** | **88Sr (V)** | **84Sr/86Sr** |
| --- | --- | --- | --- | --- | --- | --- | --- |
| Fumane 1 (N) |  |  |  |  |  |  |  |
| Outer | FUM1-02 | *0.7111* | 0.7070 | 0.00005 | 0.00097 | 1.14 | 0.05515 |
| Inner | FUM1-03 | *0.7122* | 0.7091 | 0.00016 | 0.00018 | 1.36 | 0.05644 |
| Inner | FUM1-04 | *0.7118* | 0.7090 | 0.00020 | 0.00040 | 1.51 | 0.05627 |
| Inner | FUM1-05 | *0.7137* | 0.7104 | 0.00015 | 0.00016 | 1.30 | 0.05633 |
| Inner | FUM1-06 | *0.7132* | 0.7089 | 0.00027 | 0.00025 | 1.00 | 0.05531 |
| Inner | FUM1-07 | *0.7110* | 0.7078 | 0.00011 | 0.00801 | 1.36 | 0.05578 |
|  | **Mean** |  | **0.7087** |  |  |  |  |
| Fumane 2 (M) |  |  |  |  |  |  |  |
| Outer | FUM2-01 | *0.7102* | 0.7076 | 0.00007 | 0.00014 | 1.68 | 0.05572 |
| Outer | FUM2-02 | *0.7097* | 0.7071 | 0.00004 | 0.00022 | 1.65 | 0.05518 |
| Inner | FUM2-03 | *0.7118* | 0.7102 | 0.00012 | 0.00398 | 2.75 | 0.05666 |
| Inner | FUM2-04 | *0.7118* | 0.7102 | 0.00010 | 0.00028 | 2.80 | 0.05685 |
| Inner | FUM2-05 | *0.7105* | 0.7092 | 0.00005 | 0.00032 | 3.28 | 0.05685 |
| Inner | FUM2-06 | *0.7102* | 0.7091 | 0.00004 | 0.00062 | 3.96 | 0.05678 |
| Inner | FUM2-07 | *0.7107* | 0.7095 | 0.00005 | 0.00031 | 3.59 | 0.05674 |
| Inner | FUM2-08 | *0.7108* | 0.7096 | 0.00005 | 0.00041 | 3.80 | 0.05669 |
|  | **Mean** |  | **0.7091** |  |  |  |  |
| Fumane 4 (N) |  |  |  |  |  |  |  |
| Outer | FUM4-01 | *0.7152* | 0.7117 | 0.00031 | 0.00051 | 1.21 | 0.05783 |
| Outer | FUM4-02 | *0.7132* | 0.7098 | 0.00020 | 0.00183 | 1.23 | 0.05763 |
|  | **Mean** |  | **0.7107** |  |  |  |  |
| Fumane 5 (N) |  |  |  |  |  |  |  |
| Outer | FUM5-02 | *0.7154* | 0.7106 | 0.00018 | 0.00007 | 0.88 | 0.05760 |
| Outer | FUM5-03 | *0.7135* | 0.7100 | 0.00012 | 0.00228 | 1.22 | 0.05728 |
|  | **Mean** |  | **0.7103** |  |  |  |  |
| Fumane 6 (M) |  |  |  |  |  |  |  |
| Outer | FUM6-01 | *0.7102* | 0.7077 | 0.00005 | 0.00079 | 1.78 | 0.05525 |
| Outer | FUM6-02 | *0.7106* | 0.7086 | 0.00006 | 0.00352 | 2.08 | 0.05527 |
|  | **Mean** |  | **0.7082** |  |  |  |  |

**1. Strontium isotope analysis: modern plants and snails**

The localities from which plants and snails were sampled are mapped in Figure 2 and listed in Supplementary Table 1, while results of the strontium isotope analyses on these samples are reported respectively in Supplementary Tables 4 and 5. The three sampling localities in proximity of the site (FUM-A, FUM-B, FUM-C) have a mean value of 0.7090±0.0005, which represents the strontium isotope composition of the lithology of the cave (Table 2). The average of the strontium isotope compositions of the localities within a 1km from Fumane Cave is 0.7087±0.0005, while that for the localities within 5km of the site is 0.7084±0.0006.

**S1 Table 4. Sampling location, type, corrected strontium isotope ratios and strontium concentration (Sr in parts per mil) of the modern plants analyzed.**

| **S-EVA** | **Location** | **Plant sample type** | **^87^Sr/^86^Sr corrected** | **1 SE** | **Sr (ppm)** | **Notes** |
| --- | --- | --- | --- | --- | --- | --- |
| 29671 | FUM-A | shallow-rooting | 0.7096 | 0.000017 | 13 |  |
| 29672 | FUM-A | medium-rooting | 0.7094 | 0.000007 | 501 |  |
| 29673 | FUM-A | deep-rooting | 0.7093 | 0.000007 | 1415 |  |
| 29674 | FUM-B | shallow-rooting | 0.7086 | 0.000010 | 30 |  |
| 29675 | FUM-B | medium-rooting | 0.7092 | 0.000009 | 66 |  |
| 29676 | FUM-B | deep-rooting | 0.7093 | 0.000006 | 1567 |  |
| 29677 | FUM-C | shallow-rooting | 0.7083 | 0.000009 | 60 |  |
| 29678 | FUM-C | medium-rooting | 0.7083 | 0.000012 | 48 |  |
| 29679 | FUM-C | deep-rooting | - | - | 4 | low signal |
| 29680 | FUM-D | shallow-rooting | 0.7085 | 0.000016 | 16 |  |
| 29681 | FUM-D | medium-rooting | 0.7093 | 0.000005 | 1562 |  |
| 29682 | FUM-E | shallow-rooting | 0.7091 | 0.000007 | 553 |  |
| 29683 | FUM-E | medium-rooting | 0.7091 | 0.000008 | 1070 |  |
| 29760 | FUM-E | deep-rooting | 0.7077 | 0.000006 | 97 |  |
| 29761 | FUM-F | shallow-rooting | 0.7083 | 0.000006 | 64 |  |
| 29762 | FUM-F | medium-rooting | 0.7082 | 0.000005 | 47 |  |
| 29763 | FUM-F | deep-rooting | 0.7080 | 0.000006 | 206 |  |
| 29764 | FUM-G | shallow-rooting | 0.7077 | 0.000005 | 31 |  |
| 29765 | FUM-G | medium-rooting | 0.7077 | 0.000006 | 88 |  |
| 29766 | FUM-G | deep-rooting | 0.7077 | 0.000006 | 407 |  |
| 29767 | FUM-H | shallow-rooting | 0.7090 | 0.000007 | 50 |  |
| 29768 | FUM-H | medium-rooting | 0.7079 | 0.000005 | 147 |  |
| 29769 | FUM-H | deep-rooting | 0.7080 | 0.000006 | 74 |  |
| 29770 | FUM-I | shallow-rooting | 0.7081 |  | 115 |  |
| 29771 | FUM-J | shallow-rooting | 0.7085 | 0.000006 | 25 |  |
| 29817 | FUM-J | medium-rooting | 0.7085 | 0.000008 | 53 |  |
| 29818 | FUM-J | deep-rooting | 0.7089 | 0.000008 | 26 |  |
| 29819 | FUM-J | medium-rooting | 0.7085 | 0.000005 | 20 |  |
| 29820 | FUM-J | medium-rooting | 0.7086 | 0.000006 | 40 |  |
| 29821 | FUM-J | deep-rooting | 0.7087 | 0.000006 | 34 |  |
| 29822 | FUM-K | shallow-rooting | 0.7088 | 0.000009 | 21 |  |
| 29823 | FUM-K | medium-rooting | 0.7087 | 0.000010 | 86 |  |
| 29824 | FUM-K | deep-rooting | 0.7089 | 0.000007 | 22 |  |
| 29825 | FUM-L | shallow-rooting | 0.7087 | 0.000012 | 16 |  |
| 29826 | FUM-L | medium-rooting | 0.7084 | 0.000006 | 71 |  |
| 29827 | FUM-L | deep-rooting | 0.7088 | 0.000007 | 28 |  |
| 29828 | FUM-M | shallow-rooting | 0.7080 | 0.000010 | 15 |  |
| 29829 | FUM-M | medium-rooting | 0.7082 | 0.000004 | 59 |  |
| 29830 | FUM-M | deep-rooting | 0.7082 | 0.000005 | 48 |  |
| 29831 | FUM-N | shallow-rooting | 0.7093 | 0.000008 | 28 |  |
| 29832 | FUM-N | medium-rooting | 0.7089 | 0.000006 | 51 |  |
| 29833 | FUM-N | deep-rooting | 0.7089 | 0.000007 | 53 |  |
| 29834 | FUM-O | shallow-rooting | 0.7104 | 0.000010 | 25 |  |
| 29835 | FUM-O | medium-rooting | 0.7104 | 0.000007 | 50 |  |
| 29836 | FUM-O | deep-rooting | 0.7102 | 0.000007 | 81 |  |
| 29837 | FUM-P | shallow-rooting | 0.7095 | 0.000005 | 50 |  |
| 29838 | FUM-P | medium-rooting | 0.7098 | 0.000005 | 220 |  |
| 29839 | FUM-P | deep-rooting | 0.7097 | 0.000005 | 30 |  |
| 29840 | FUM-PA | shallow-rooting | 0.7089 | 0.000007 | 258 |  |
| 29841 | FUM-PA | medium-rooting | 0.7052 | 0.000005 | 948 |  |
| 29842 | FUM-PA | deep-rooting | 0.7052 | 0.000006 | 266 |  |
| 29843 | FUM-Q | shallow-rooting | 0.7095 | 0.000006 | 239 |  |
| 29844 | FUM-Q | medium-rooting | 0.7095 | 0.000007 | 239 |  |
| 29845 | FUM-Q | deep-rooting | - | - | - | saturated |
| 29846 | FUM-R | shallow-rooting | 0.7076 | 0.000005 | 46 |  |
| 29847 | FUM-R | medium-rooting | 0.7079 | 0.000006 | 185 |  |
| 29848 | FUM-R | deep-rooting | 0.7077 | 0.000006 | 260 |  |

**S1 Table 5.** Strontium isotope ratios for modern terrestrial mollusc shells. Sampling location (for more information see Fig. 2 and Supplementary Table 1), taxon analyzed, corrected ^87^Sr/^86^Sr ratios and strontium concentration (Sr in parts for mil) is included.

| **S-EVA** | **Location** | **Sample type** | **^87^Sr/^86^Sr corrected** | **1 SE** | **Sr (ppm)** |
| --- | --- | --- | --- | --- | --- |
| 29615 | FUM-A | *P. elegans* | 0.7089 | 0.000010 | 36 |
| 29616 | FUM-B | *P. elegans* | 0.7089 | 0.000010 | 19 |
| 29617 | FUM-C | *P. elegans* | 0.7077 | 0.000008 | 83 |
| 29618 | FUM-D | *P. elegans* | 0.7077 | 0.000008 | 136 |
| 29619 | FUM-E | *P. elegans* | 0.7086 | 0.000005 | 432 |
| 29620 | FUM-F | *P. elegans* | 0.7076 | 0.000006 | 241 |
| 29621 | FUM-G | other gastropod | 0.7075 | 0.000006 | 348 |
| 29622 | FUM-H | *P. elegans* | 0.7077 | 0.000009 | 173 |
| 29623 | FUM-J | *P. elegans* | 0.7080 | 0.000010 | 53 |
| 29624 | FUM-K | *P. elegans* | 0.7081 | 0.000007 | 80 |
| 29625 | FUM-L | *P. elegans* | 0.7077 | 0.000007 | 46 |
| 29626 | FUM-M | *P. elegans* | 0.7078 | 0.000008 | 67 |
| 29627 | FUM-N | *P. elegans* | 0.7081 | 0.000006 | 140 |
| 29628 | FUM-O | other gastropod | 0.7094 | 0.000007 | 145 |
| 29629 | FUM-P | *P. elegans* | 0.7089 | 0.000007 | 120 |
| 29630 | FUM-PA | *P. elegans* | 0.7056 | 0.000007 | 386 |
| 29631 | FUM-R | *P. elegans* | 0.7077 | 0.000009 | 284 |

**S1 Table 6.** **Strontium isotope ratios of archaeological common vole (*Microtus arvalis*) dental enamel prepared in solution.** Information on stratigraphic unit of origin, corrected ^87^Sr/^86^Sr ratios and strontium concentration (Sr in parts per million) is included.

| **Stratigraphic unit** | **S-EVA** | **^87^Sr/^86^Sr corrected** | **Sr (ppm)** |
| --- | --- | --- | --- |
| A2 | 29599 | 0.7091 | 95 |
| A2 | 29600 | 0.7093 | 75 |
| A2 | 29601 | 0.7098 | 101 |
| A2 | 29602 | 0.7097 | 77 |
| **A2** | **mean A2** | **0.7095 ± 0.0003** |  |
| A3 | 29603 | 0.7101 | 119 |
| A3 | 29604 | 0.7100 | 98 |
| A3 | 29605 | 0.7097 | 100 |
| A3 | 29606 | 0.7098 | 82 |
| **A3** | **mean A3** | **0.7099 ± 0.0002** |  |
| A9 | 29607 | 0.7110 | 126 |
| A9 | 29608 | 0.7094 | 54 |
| A9 | 29609 | 0.7089 | 65 |
| A9 | 29610 | 0.7087 | 176 |
| **A9** | **mean A9** | **0.7095 ± 0.0010** |  |
| A11 | 29611 | 0.7094 | 91 |
| A11 | 29612 | 0.7102 | 95 |
| A11 | 29613 | 0.7094 | 88 |
| A11 | 29614 | 0.7095 | 99 |
| **A11** | **mean A11** | **0.7096 ± 0.0004** |  |

**2. Hominin teeth**

The human teeth analyzed as part of this study, illustrated schematically in Supplementary Fig. 1 and listed in Table 1, are here described in detail from the oldest to the most recent and the results reported in Supplementary Table 3.

Fumane 1 is a lower left second deciduous molar (Ldm_2_) assignable to Neanderthals based on its lateral aspect and enamel-dentine junction features^1^. The degree of root resorption of this tooth (stage Res¾)^2^ corresponds to an age ranging from about 9 to 12 years, based on the work by Shackelford et al.^3^. In modern humans, the cusps of such teeth start forming a few weeks before birth and the crown is completely formed between 10.5 months and 1.5 years after birth^5^. This tooth starts erupting at about 1.5 years and is fully erupted by 2.5 years.

Fumane 4 is an upper right central deciduous incisor (Rdi^1^) that cannot be attributed with certainty on morphological grounds either to Neanderthal*s* or to modern humans, even though the Z-score computed for its bucco-lingual diameter is closer to the latter human taxon^1^. The stage of resorption of Fumane 4 is close to grade Res ¾ of Moorrees et al.^2^, suggesting that the tooth had been lost ante-mortem through dental development, a stage that corresponds to an age of approximately 6 years in recent modern children^4,5^. In modern humans, the crown of upper central deciduous incisors starts forming during intrauterine life and is fully formed at birth, or at latest 1.5 months after birth; it starts erupting at about 4.5 months and is fully erupted at 10.5 months^4^.

Fumane 5 is a lower right lateral deciduous incisor (Rdi_2_) that based on its faint mesial and distal ridges on the lingual aspect, as well as on the large bucco-lingual diameter, can be attributed to a Neandertal^2^. The preserved root stump, more elongated labially (6.05 mm) than lingually (1.45 mm), appears to be resorptive, confirming an age of approximately 6 years on the basis of recent human standards^4,5^. In modern humans, the crown forms during intrauterine life and is almost complete at birth, or at latest 4.5 months after birth; crown eruption starts at about 7.5 months and is completed by 1.5 years^4^. As Fumane 4 and Fumane 5 were recovered in the same unit and as they are at the same, final, stage of resorption it is likely that they belong to the same individual, a Neandertal of approximately 6 years of age based on recent *H. sapiens* standards^1^.

Fumane 6 is a small permanent lower molar fragment with a short segment of root, which has proven impossible to attribute taxonomically. Its wear stage, however, clearly indicates that it belonged to a different individual than those whose teeth were found below and above it. Not knowing what tooth this fragment belonged to we cannot indicate at what time the crown may have formed. The earliest estimate would be that for a first molar, the crown of which starts forming during the first year of life and is fully formed between the second and third year of life^5^. The eruption of first molars generally occurs between 5.5 and 6.5 years of age, with both amelogenesis and eruption being significantly later in the case of second and third molars.

Fumane 2 is an upper right lateral deciduous incisor (Rdi^2^), which cannot be assigned to species morphologically, but was genetically-typed as *H. sapiens*^6^. The short segment of the root, longer labially (6.1 mm) than lingually (0.8 mm), looks resorptive, indicating an age of ~6/7 years, on the basis of recent human standards^4,5^. In upper lateral deciduous incisors, the crown forms during intrauterine life and is almost complete at birth, or at latest 4.5 months after birth, and it starts erupting at about 7.5 months and is fully erupted by 1.5 years of age^4^.

**S1 Figure 1. Schematic drawings of the human teeth sampled for this study with the areas that were laser ablated.** All specimens were preferentially sampled in the middle-to-upper portions of the dental crowns.

**Supplementary Information References**

1. Benazzi, S. *et al*. Middle Palaeolithic and Uluzzian human remains from Fumane Cave, Italy. *J. Hum. Evol.* **70**, 61-68 (2014).
2. Moorrees, C.F.A., Fanning, E.A. & Hunt, Jr. E.E. Formation and resorption of three deciduous teeth in children. *Am. J. Phys. Anthropol.* **21**, 205–213 (1963).
3. Shackleford, L.L., Stinespring Harris, A.E. & Konigsberg, L.W. Estimating the distribution of probable age-at-death from dental remains of immature human fossils. *Am. J. Phys. Anthropol.* **147**, 227-253 (2012).
4. AlQahtani, S.J., Hector, M.P. & Liversidge, H.M. The London atlas of human tooth development and eruption. *Am. J. Phys. Anthropol.* **142**, 481–490 (2010).
5. Ubelaker, D.H. *Human Skeletal Remains. Excavations, Analysis, Interpretation* (Aldine, Chicago, 1978).
6. Benazzi, S. *et al*. The makers of the Protoaurignacian and implications for Neandertal extinction. *Science* **348**, 793-796 (2015).
