# Supplementary figures and images for: Strontium isotope evidence for Neanderthal and modern human mobility at the upper and middle palaeolithic site of Fumane Cave (Italy)
Source: PLoS One. 2021 Aug 24;16(8):e0254848. doi: 10.1371/journal.pone.0254848 (PMC8384160; doi:10.1371/journal.pone.0254848)

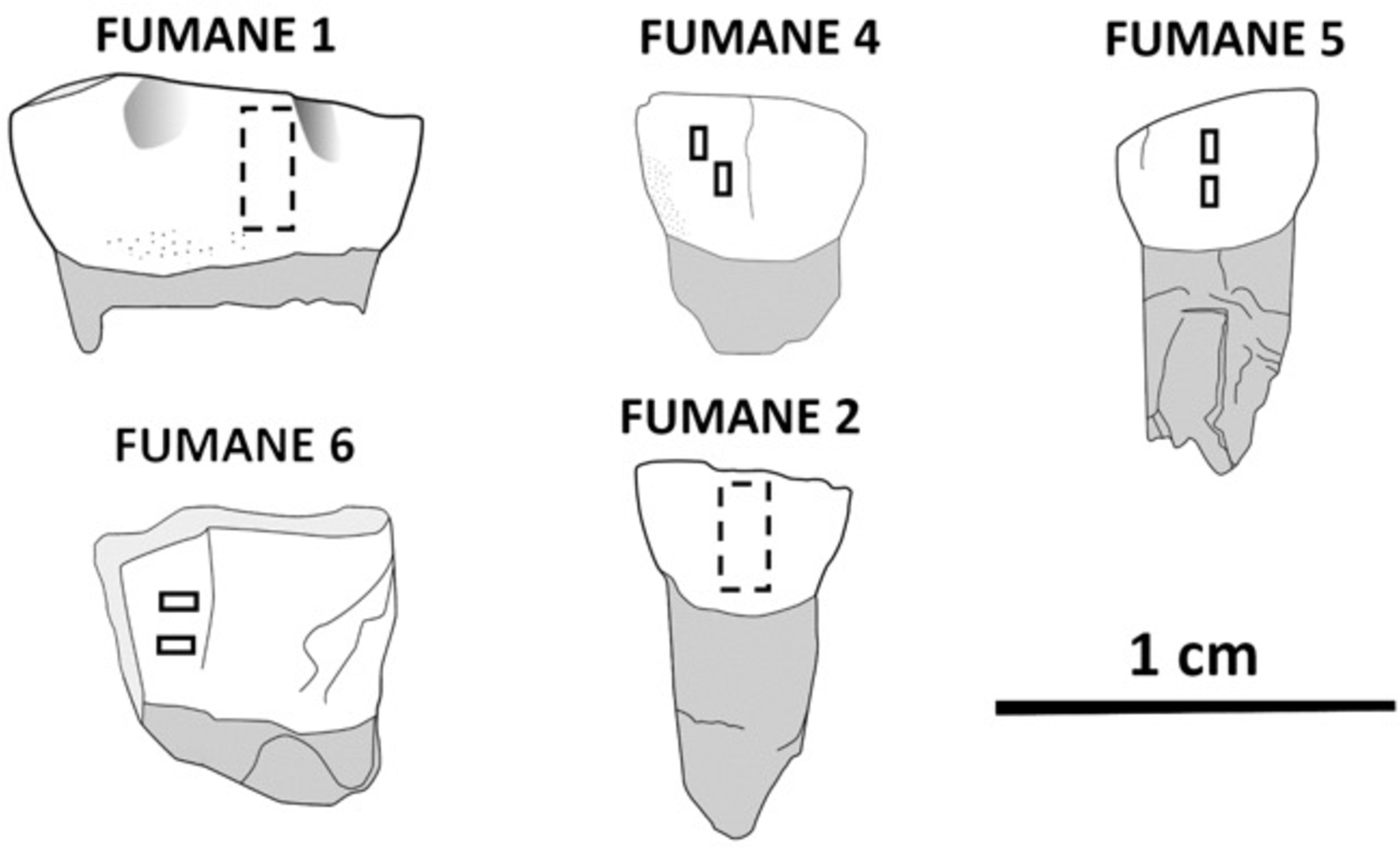

Supplement: S1 Fig — (TIF) [file pone.0254848.s002.tif]
